# Profiling Trait Anxiety: Transcriptome Analysis Reveals Cathepsin B (*Ctsb*) as a Novel Candidate Gene for Emotionality in Mice

**DOI:** 10.1371/journal.pone.0023604

**Published:** 2011-08-29

**Authors:** Ludwig Czibere, Laura A. Baur, Anke Wittmann, Katja Gemmeke, Andrea Steiner, Peter Weber, Benno Pütz, Nafees Ahmad, Mirjam Bunck, Cornelia Graf, Regina Widner, Claudia Kühne, Markus Panhuysen, Boris Hambsch, Gabriele Rieder, Thomas Reinheckel, Christoph Peters, Florian Holsboer, Rainer Landgraf, Jan M. Deussing

**Affiliations:** 1 Max Planck Institute of Psychiatry, Munich, Germany; 2 Helmholtz Zentrum München, Institute of Developmental Genetics, Neuherberg, Germany; 3 Affectis Pharmaceuticals, Martinsried, Germany; 4 Max von Pettenkofer Institute, Ludwig Maximilians University, Munich, Germany; 5 Institute of Molecular Medicine and Cell Research, Faculty of Biology, Albert Ludwigs University, Freiburg, Germany; Instituto Nacional de Câncer, Brazil

## Abstract

Behavioral endophenotypes are determined by a multitude of counteracting but precisely balanced molecular and physiological mechanisms. In this study, we aim to identify potential novel molecular targets that contribute to the multigenic trait “anxiety”. We used microarrays to investigate the gene expression profiles of different brain regions within the limbic system of mice which were selectively bred for either high (HAB) or low (LAB) anxiety-related behavior, and also show signs of comorbid depression-like behavior.

We identified and confirmed sex-independent differences in the basal expression of 13 candidate genes, using tissue from the entire brain, including coronin 7 (*Coro7*), cathepsin B (*Ctsb*), muscleblind-like 1 (*Mbnl1*), metallothionein 1 (*Mt1*), solute carrier family 25 member 17 (*Slc25a17*), tribbles homolog 2 (*Trib2*), *z*inc finger protein 672 (*Zfp672*), syntaxin 3 (*Stx3*), ATP-binding cassette, sub-family A member 2 (*Abca2*), ectonucleotide pyrophosphatase/phosphodiesterase 5 (*Enpp5*), high mobility group nucleosomal binding domain 3 (*Hmgn3*) and pyruvate dehydrogenase beta (*Pdhb*). Additionally, we confirmed brain region-specific differences in the expression of synaptotagmin 4 (*Syt4*).

Our identification of about 90 polymorphisms in *Ctsb* suggested that this gene might play a critical role in shaping our mouse model's behavioral endophenotypes. Indeed, the assessment of anxiety-related and depression-like behaviors of *Ctsb* knock-out mice revealed an increase in depression-like behavior in females.

Altogether, our results suggest that *Ctsb* has significant effects on emotionality, irrespective of the tested mouse strain, making it a promising target for future pharmacotherapy.

## Introduction

Most behavioral patterns are shaped by both genetic and environmental influences. Genetic factors first determine a set but flexible framework, and then environmental influences fix the respective behavior within the genetically given constraints. These two influences appear to be the most important actors in the developmental process. Thus, some of us are predisposed to never develop any kind of psychiatric disorder, whereas others might succumb from only minor external stimuli [1], [2], [3].

Multiple mechanisms that increase anxiety have evolved in the animal kingdom [4]. Since these systems are very old in evolutionary terms, many other molecular systems have since evolved to control anxiety, closely connected stress-induced physiological responses, and to re-establish a homeostatic state afterwards. Twin studies of anxiety and depression enable scientists to estimate the heritability of psychiatric disorders, with estimates for the heritability of major depression and phobia spectrum disorders ranging from 28 to 60%. These studies also show increased prevalence of these disorders within some families, while others are completely unaffected [5], [6], [7], [8]. Since approximately 40% of the population of industrialized countries is at risk to develop some kind of anxiety or mood disorder [9], the costs of treatment and economic losses caused by these disorders are large and steadily increasing. The mechanisms underlying the etiology of these disorders are not well characterized, and the efficacy of current therapies is not high enough to result in full recovery of many patients. These facts underline the urgent need for new, more effective therapeutic methods with fewer side effects.

Since psychiatric and behavioral traits are linked to a wide variety of genomic regions, with each region contributing less than 10% to the total phenotypic variation (quantitative trait locus), it is important to screen a large number of loci to reveal the impact of a single genomic locus. The key problem of quantitative trait locus analysis for complex psychiatric disorders is that, in most cases, linkage or association studies deal with rather small effect sizes that are difficult to reproduce in human studies, and low marker densities are common in animal studies [10], [11], [12]. For the latter, the identified regions may contain many genes, and finer mapping usually requires a large expenditure of time and money.

One alternative approach that can still identify the genes involved in shaping a specific phenotype is gene expression profiling. Since current technology cannot characterize patients by the molecular biological processes in their brains, the best way to explore these mechanisms is through animal models. Since the exonic genome parts of rodents are approximately 92% homologous to those of humans, rodents with their short reproduction time are prime candidates for use in these models [13], [14].

The high (HAB) and low (LAB) anxiety-related behavior mouse model, used to analyze the traits of anxiety and depression, has been well described and characterized. It has also been validated in a variety of behavioral tests, which assess both anxiety-related (such as elevated plus-maze – EPM, light/dark box test, ultra sonic vocalization – USV) and depression-like (tail-suspension test – TST, forced swimming test – FST) behaviors. As a further validation, the administration of diazepam results in a decrease in measured anxiety-related behavior in the USV in HAB, but not LAB, mice [14], [15]. Some genes have already been proposed as contributors to the observed phenotypes in this model, including arginine vasopressin-neurophysin II-copeptin *(Avp)*, which is significantly less expressed in LAB mice compared to HAB and wildtype CD-1 mice. Importantly, LAB mice have been identified as carrying a single-nucleotide polymorphism homozygously in the *Avp* gene, as well as a deletion of 12 bp in the promoter region. Either of these could lead to a reduction in gene expression or mRNA processing, resulting in a decreased amount of the peptide, and ultimately causing a 50% reduction in bioactive AVP and a non-grave variant of familial *diabetes insipidus* [16], [17]. Similar findings also exist for glyoxalase 1 (*Glo1*), enolase phosphatase (*Enoph1*) and the transmembrane protein 132 D (*Tmem132d*) [15], [18], [19], [20].

Thus, the HAB/LAB model, with its strongly genetically fixed background, is ideal for deeper molecular characterization. As identification of differential gene and protein expression between both lines has revealed more gene sequence-based differences [19], a comprehensive screening on gene expression had become more important. Therefore, we focused on the emotion-regulating parts of the limbic system and the brain regions closely connected to them: the hypothalamic paraventricular nucleus (PVN), the supraoptic nucleus (SON), the basolateral (BLA) and central (CeA) amygdala, and the cingulate cortex (Cg), as well as the *nucleus accumbens* (NAc) and dentate gyrus (DG). Our previous results support the hypothesis that these regions are critical in producing the respective endophenotypes in HAB/LAB mice [21].

To gain insight into differences at the gene expression level that could underlie the observed anxiety-related endophenotypes, we conducted a comprehensive overview of gene expression in these brain regions, utilizing the MPIP24k microarray and the MouseWG-6 v1.1 Expression BeadChip-system (Illumina, San Diego, CA), which provided information from about 45,000 unique probes. As gene expression data from microarray analyses are generally not seen as being stably reproducible, these results were further confirmed by quantitative polymerase chain reaction (qPCR), and partial confirmation was obtained by *in situ* hybridization analyses. We further expanded our analyses by looking at the underlying DNA sequences, and finally characterized a knock-out (KO) mouse for Cathepsin B (*Ctsb*), the candidate gene that showed the most prominent variations throughout our data.

## Results

### Microarray, qPCR and *in situ* hybridization

**Figure 1:**
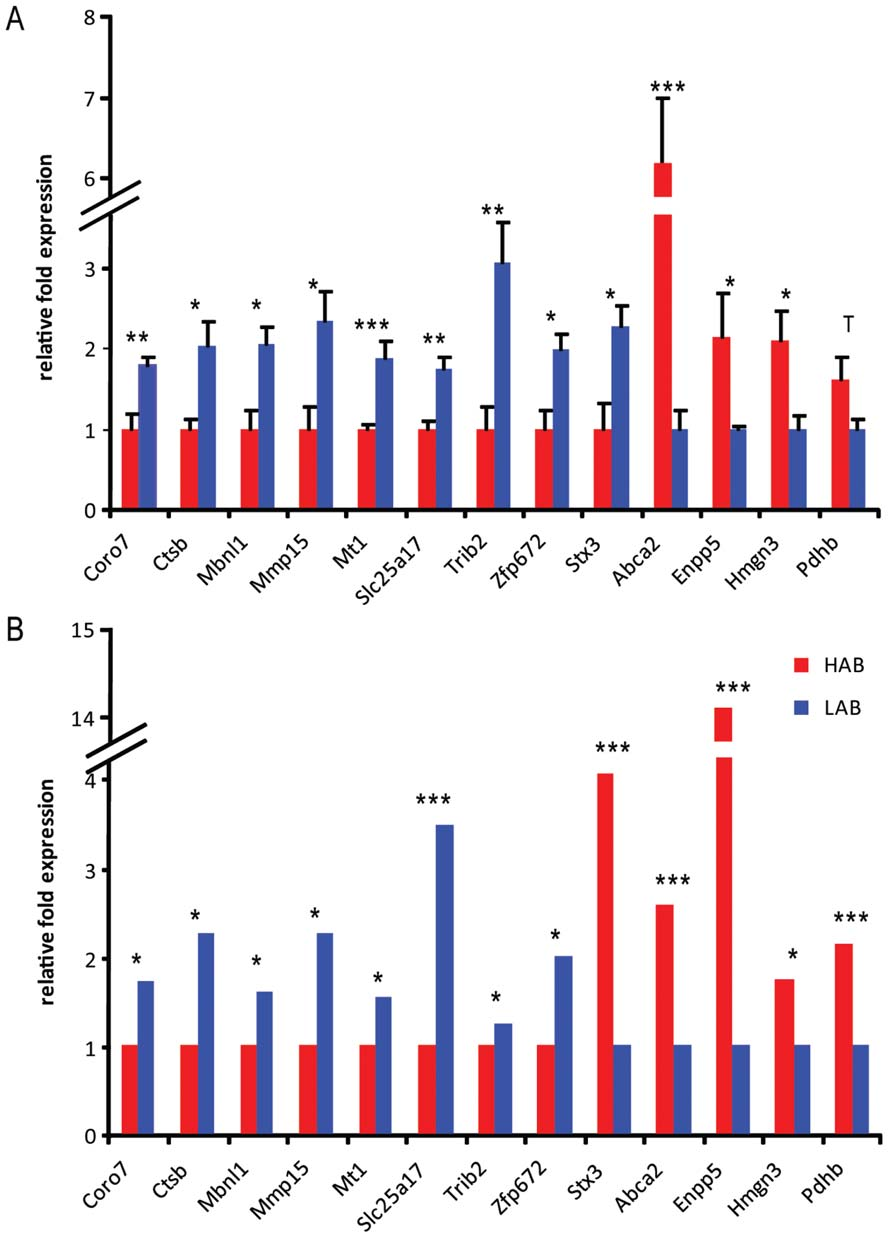
Gene expression profiles of HAB *vs.* LAB mice in multiple brain regions. (A) gene expression confirmed by quantitative PCR and (B) as detected by microarray-based gene expression analysis. N = 6–10 per group. Data are presented as means and for (A) +SEM; T p<0.1, * p<0.05, ** p<0.01, *** p<0.001.

**Figure 2:**
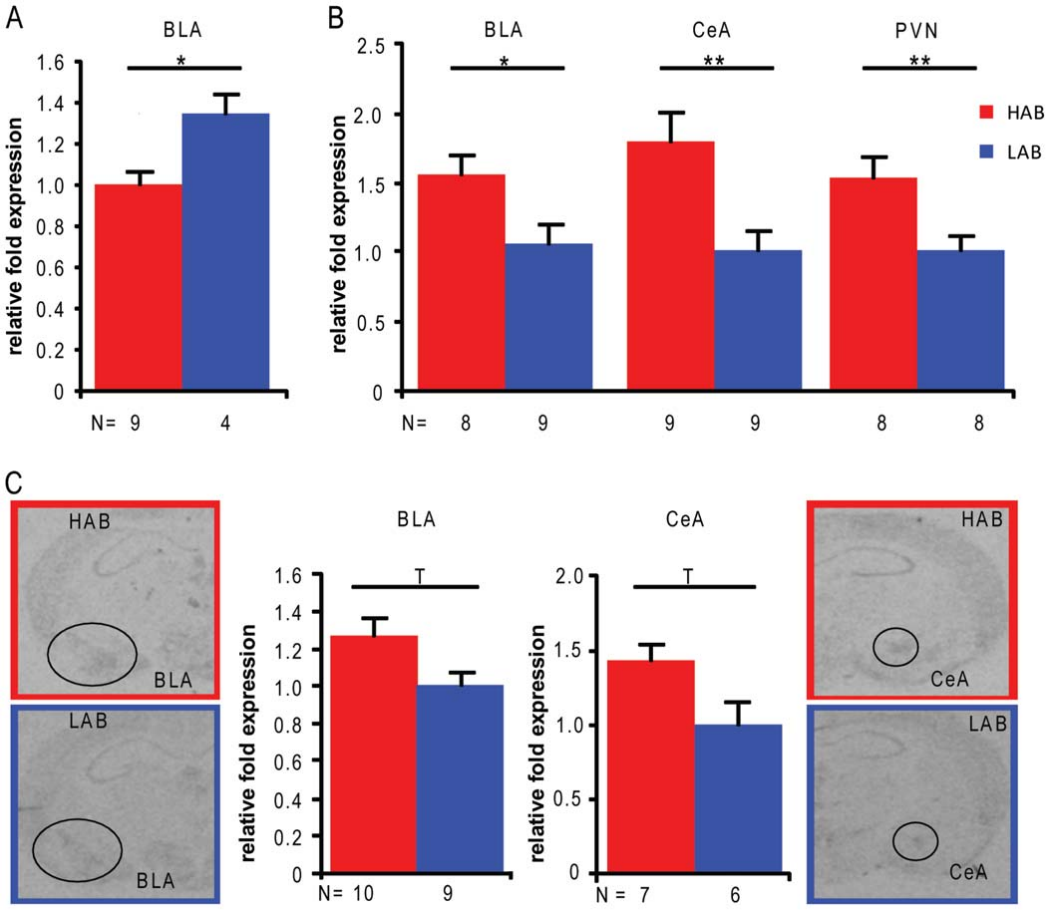
Region-specific gene expression in HAB *vs.* LAB mice. Expression (A) of Syntaxin3 (*Stx3*) in the basolateral amygdala (BLA) and (B) of synaptotagmin 4 (*Syt4*) in the BLA, the central amygdala (CeA) and the hypothalamic paraventricular nucleus (PVN) as measured by quantitative PCR and (C) *Syt4* quantified from *in sit*u hybridization. Data are presented as means +SEM; T p<0.1, * p<0.05, ** p<0.01.

In a negative control experiment, hybridizing identical dye-swapped samples from the MPIP24k arrays did not return a single statistically significant result. From all MPIP24k and Illumina platform-based microarray experiments, we found over 300 candidate genes which were differentially regulated between HAB and LAB mice in all analyzed brain regions. All data are available in the NCBI GEO database under the accession number GSE29015. From these, the top 32 candidates were investigated in the follow-up qPCR experiment and other analyses (Table S1, excerpt Fig. 1B). In every experiment (that is, throughout all brain regions), ATP-binding cassette, sub-family A member 2 *(Abca2)*, *Ctsb*, ectonucleotide pyrophosphatase/phosphodiesterase 5 *(Enpp5)*, and tau tubulin kinase 1 *(Ttbk1)* displayed the largest difference in regulation between lines (>500%). Other genes also attracted our attention, like transthyretin *(Ttr)* in the BLA and synaptotagmin 4 *(Syt4)* in the PVN, CeA and BLA. These were oppositely regulated between our two lines, but only in single brain regions. However, we focused on the other genes for this study, as we hypothesized that changes in regulation between most brain regions are more likely to represent general systemic changes, and are therefore more likely to be associated with changes in the phenotypes under consideration. We were able to confirm differences in gene regulation for 13 of the selected genes, by using qPCR on mRNA from the entire brain, with twelve of the candidates delivering statistically significant results (p<0.05; Figure 1A). Although the results for syntaxin 3 *(Stx3)* showed an opposite effect in the microarray experiments (higher in HAB than in LAB mice) compared to the qPCR, we can rely on the data of the qPCR, as the qPCR data has been replicated a second time with independent samples from the BLA (Fig. 2A). The relatively high number of SNPs detected in the 15^th^ exon of *Stx3* (Table S2) could also explain the higher affinity of *Stx3* probes for the HAB-specific mRNA in the microarray experiment. For most genes, the difference in regulation is independent of sex, as the microarray analyses were performed with male mice, and the validation by qPCR was performed with male mice as well as female mice. Thus, for the genes coronin 7 *(Coro7)*, *Ctsb*, muscleblind-like 1 *(Mbnl1)*, matrix metallopeptidase 15 *(Mmp15)*, *Mt1*, solute carrier family 25 member 17 *(Slc25a17)*, tribbles homolog 2 *(Trib2)*, *z*inc finger protein 672 *(Zfp672)* and *Stx3*, differential expression could be demonstrated irrespective of sex in HAB *vs.* LAB mice.

Differential expression of *Ttr* in the BLA could not be confirmed, in contrast to *Syt4*, where qPCR (Fig. 2B) and *in situ* hybridization (Fig. 2C) detected significantly higher expression in the PVN, CeA and BLA of HAB compared to LAB mice.

### Sequencing

**Figure 3:**
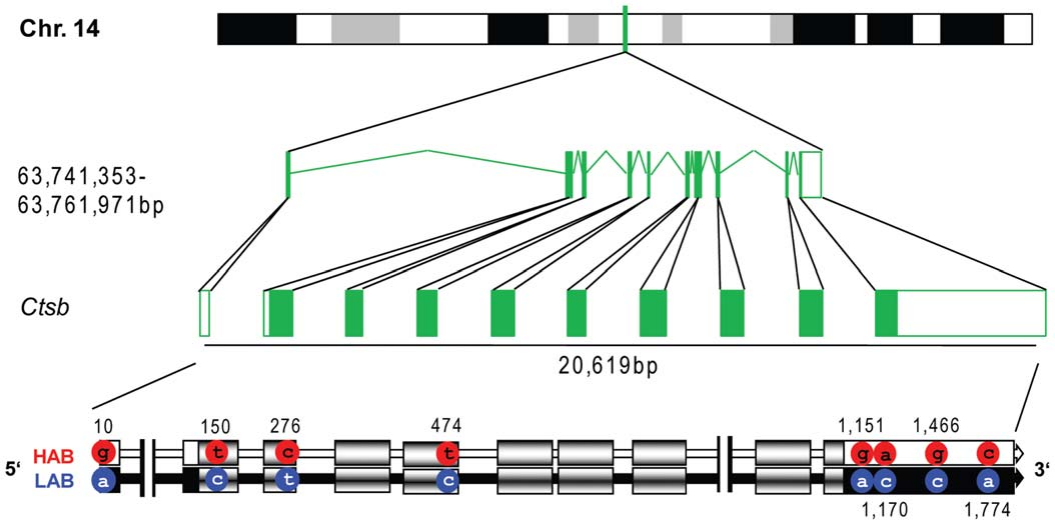
Cathepsin B *(Ctsb)* gene sequence of HAB *vs.* LAB mice. Polymorphic sites are shown with positions in the coding sequence referenced to the transcription start in the spliced mRNA in bp. Exons and untranslated regions (UTRs) are indicated by boxes (exons shaded, UTRs completely filled black or white).

We used sequencing to identify possible molecular correlates of differences in gene expression between HAB and LAB mice. Analysis of the HAB and LAB *Mt1* locus failed to reveal any genetic differences. In contrast, many polymorphic loci were identified at the *Ctsb* locus. Here, we found 76 SNPs, eight insertions and nine deletions in HAB *vs.* LAB mice (defining insertion and deletion relative to the mouse strain C57BL/6J). In the promoter region, ten SNPs and two insertions were found. In these ten exons, eight polymorphic sites (all SNPs) were identified (Fig. 3). The vast majority of variations were found in the introns and the downstream enhancer region (Table S3). Interestingly, about six variations are located within a single 230 bp sequence (between −2,269 and −2,045 bp in the promoter). A similar density of polymorphic sites was found in the third and fourth intron, where 18 variations were found in a 550 bp sequence, and 12 additional variations were found in another 400 bp sequence. Additionally, condensation of twelve variable sites could be identified in the downstream enhancer region (at a length of 350 bp).

Sequencing of the primer binding sites of the genes we analyzed with qPCR did not reveal any polymorphisms, thus validating the qPCR results. The identified SNPs in the respective sequences are summarized in Table S2. Notably, the ones in *Hmgn3* cause amino acid substitutions.

### Behavioral testing of KO mice

We could not identify any significant differences in bodyweight between the *Ctsb* ^+/+^ wildtype (WT), *Ctsb* ^+/−^ heterozygous (HET) and their *Ctsb* ^−/−^ KO littermates, for both male and female mice (data not shown). The average weight difference between male and female mice was 5–6 g.

**Figure 4:**
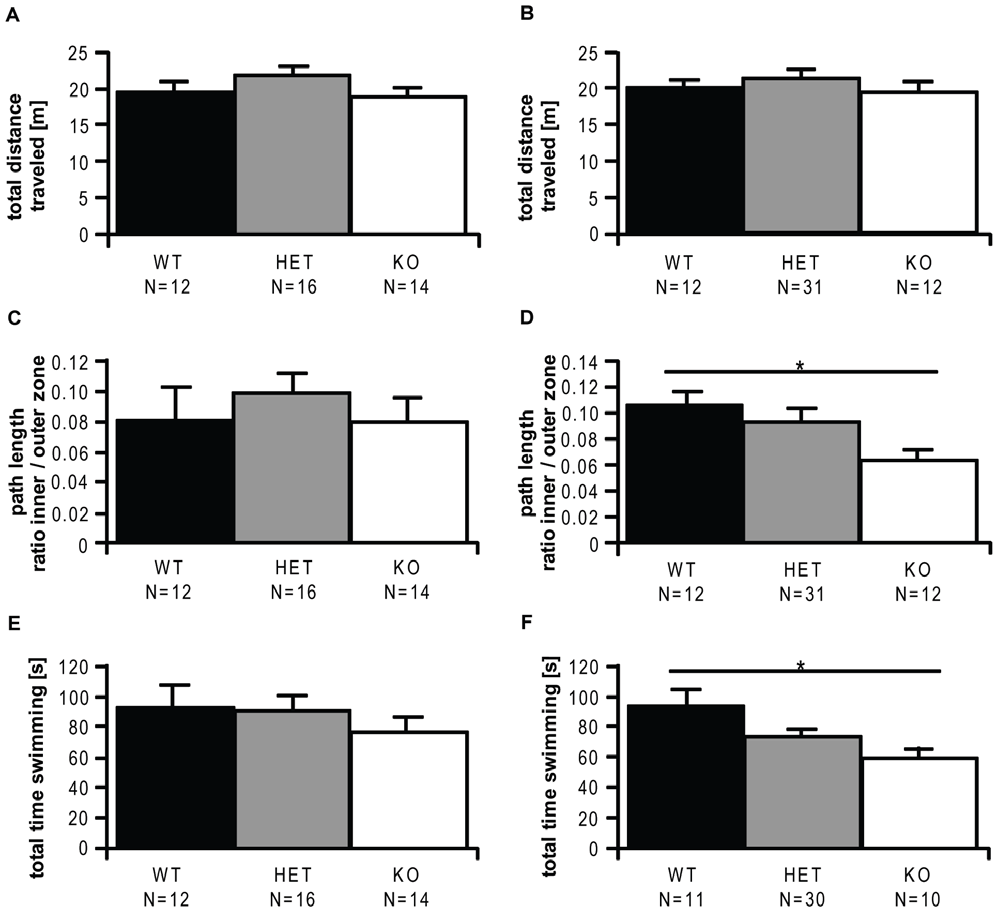
Phenotypes of Cathepsin B (*Ctsb*) knockout (KO) *vs.* heterozygous (HET) and wildtype (WT) mice. (A) Locomotion of male and (B) female mice in the open field, (C) anxiety-related behavior of male and (D) female mice as measured in the open field; (E) depression-like behavior of male and (F) female mice as reflected by the forced swim test. Data are presented as means +SEM; * p<0.05.

With respect to locomotion, there were no significant differences between any of the groups, as measured with the EPM (all arm entries, distance traveled) and the open field (OF; distance traveled; Fig. 4A and B). None of the other parameters assessed on the EPM revealed significant differences (data not shown). The data obtained from the TST were not suitable for analysis – all groups showed climbing behavior, so an evaluation regarding immobility parameters was not possible. In the OF, the different groups of male mice failed to show significant differences in the anxiety parameters (Fig. 4C), but female *Ctsb* KO mice showed a significantly decreased inner *vs.* outer zone path length ratio (Fig. 4D), combined with a decreased time spent with path length in the inner zone of the test field, reaching a statistical trend (Kruskal-Wallis H test – KWH: p<0.1, corrected Mann-Whitney U test – MWU: p<0.1 only for the WT *vs.* KO comparison; mean time spent in the inner zone ± SEM for KO: 7.6±1.0 s, HET: 11.5±1.2 s, WT: 13.0±1.7 s; mean path traveled in the inner zone ± SEM for KO: 1.2±0.2 m, HET: 1.8±0.2 m, WT: 1.9±0.2 m).

In male mice, a trend towards decreased exploratory drive, increased self-grooming and increased arousal was observed on the elevated platform (EPF), as also indicated by the increased gut activity in the *Ctsb* KO males compared to wildtype mice (KWH: p<0.1; mean grooming frequency±SEM for KO: 1.6±0.3, HET: 2.7±0.5, WT: 1.4±0.5; mean frequency of exploratory head-dips±SEM for KO: 30.8±2.8, HET: 43.1±3.6, WT: 35.8±1.9; mean frequency of defecation±SEM for KO: 4.4±0.6, HET: 3.1±0.5, WT: 2.5±0.5). Female mice did not show any significant difference.

Also, no differences were observed after stress exposure in the stress-reactivity test (SRT; data not shown).

Although no significant differences could be observed between WT and KO male mice (Fig. 4E) in the FST, several parameters showed significant differences when comparing female mice. Reduced swimming time indicated a less active coping strategy of *Ctsb* KO mice (Fig. 4F). A statistical trend was observed for the time spent floating in the same direction (KWH: p<0.1, corrected MWU: p<0.1 for the WT *vs.* KO and HET *vs.* WT comparisons; mean time floating±SEM for KO: 244±6.2s, HET: 241±4.8 s, WT: 215±11.1 s).

## Discussion

By comparing the HAB *vs.* LAB transcriptomes using microarray-based gene expression profiling, we succeeded in identifying 32 candidate genes that were consistently differently expressed between the two mouse lines. After validation of the results by qPCR, we focused on genetic variations between the two lines in the validated genes, and were able to identify about 90 variations within the *Ctsb* gene, in contrast to the *Mt1* gene, where no polymorphisms were identified. Although none of the observed SNPs in the coding sequence caused changes in the amino acid-coding triplets, they can induce changes in mRNA stability. Finally, by testing *Ctsb* KO mice and their wildtype littermates, we could demonstrate that the KO and HET alleles resulted in modestly increased anxiety-related behavior in female mice, in accordance with the decreased expression in HAB mice. Similarly, the *Ctsb* KO and HET alleles had a robust effect on depression-like behavior in the FST. Thus, our data support the hypothesis that *Ctsb* influences depression-like behavior in HAB mice.

The differential expression of 16 genes could be confirmed, even partially independent of gender. This increases our interest in the corresponding candidate genes for anxiety, as both males and females of each mouse line display the line-specific phenotypes. The candidate genes for which differential expression in the entire brain was confirmed are: *Ctsb*, *Coro7*, *Mmp15*, *Slc25a17*, *Zfp672*, *Abca2*, *Enpp5*, high mobility group nucleosomal binding domain 3 *(Hmgn3)* and pyruvate dehydrogenase beta *(Pdhb)*, *Mt1*, *Stx3*, *Mbnl1* and *Trib2*. *Syt4*, *Tmem132d* and *Avp* were also confirmed by qPCR (the latter two have been described before [16], [20]), though their differential expression was restricted to specific brain areas. We could reproduce about 50% of our gene expression levels results through microarray analysis, which seems to be acceptable, although hypotheses of region-specific gene expression differences cannot be excluded for the genes not validated.

Regarding the identified candidate genes, other studies using KO strategies have already shown that *Abca2* and *Syt4* make significant contributions to phenotypes closely related to the endophenotypes we focus on. *Abca2* KO mice showed a decrease in body weight and decreased locomotor abilities, as well as an increased susceptibility to environmental stress [22]. The last point especially suggests parallels to LAB mice, as qPCR also confirmed that the LAB mice only expressed <20% of the *Abca2* mRNA when compared to HAB mice.

Synaptotagmins, which comprise a large family of proteins that regulate vesicle trafficking in neurons, are widely considered the presynaptic “calcium sensor” in neuronal exocytosis [23], [24], [25], and are highly evolutionary conserved. *Syt4* KO mice, in comparison to WT animals, showed reduced anxiety- and depression-like behaviors in various tests, as well as enhanced locomotion in the OF [26]. This finding supports results obtained from our HAB and LAB mice, which showed increased expression of this gene in the CeA, BLA and the PVN of HAB mice. Thus, it appears likely that *Syt4* contributes to the behavioral differences observed in our animal model, and it has to be considered as another candidate gene, which might influence emotionality [25], [27], [28], [29].

The *Coro7* gene transcript and its protein have been shown to be important in brain development [30], [31]. *Mmp15* and its gene product are not well characterized with respect to brain functions, but they seem to play a role in inflammation- and oncogenesis-related processes [32], [33]. *Slc25a17* is a peroxisomal ATP transporter [34], which makes this candidate gene an interesting player in neurometabolism. The zinc finger protein *Zfp672* is a putative transcription factor that has been identified as differentially expressed between HAB and LAB mice. Similarly, *Hmgn3* was found to be 2-fold overexpressed in HAB relative to LAB mice. The two SNPs identified (rs13474367 and rs13474366) are non-synonymous coding. The first one – in all known transcript variants – represents a substitution of serine in HAB by proline in LAB mice, while the second one would only affect the amino acid sequence derived from two transcript variants, changing glutamic acid to lysine in LAB mice. Generally, HMGN family proteins significantly contribute to the differential expression of many genes [35]. *Pdhb* deficiency caused by mutations has been found to cause severe damage to amino acid neurometabolism in humans [36].

Darios and Davletov [37] described the importance of *Stx3*, which encodes a membrane protein required for neurite growth and neural development as an activator of SNARE complexes. Arachidonic acid also plays an important role in STX3 action, highlighting the impact of metabolism as a potential cofactor fundamental to neurometabolism, and therefore brain function [37]. In HAB *vs.* LAB mice, expression differences in *Stx3* have been identified, again pointing to basic differences in neuronal function between the two mouse lines (as also demonstrated here by *Syt4*, *Pdhb* and *Slc25a17*), which have the potential to cause the observed phenotypic differences.

*Enpp5* encodes a nucleotide-metabolizing ecto-enzyme, a class of enzymes that regulate the availability of extracellular nucleotides, and therefore control signaling through purinoceptors such as the P2X ion channels. Interestingly, one P2X ion channel (P2RX7) has been identified by a number of studies as having polymorphic sites (SNPs), which are associated with bipolar disorder and major depressive disorder in a large number of patients [38], [39], [40], [41]. Thus, P2RX7 or a modulator of its substrate, *Enpp5*, could be interesting novel targets for therapeutic intervention.

It has been demonstrated that neurodegenerative diseases, as well as metabolic stress, enhance the expression of *Mt1* in perivascular regions of the cerebral cortex, predominantly in astrocytes [42]. Further experiments highlighted a connection between physical stress and increased *Mt1* expression in a variety of brain regions. Increased expression has also been observed upon subcutaneous administration of steroid hormones [43], [44]. Accordingly, *Mt1* might play a key role in the HAB/LAB mouse model, where, besides differential expression, an increased stress response to physical stressors, i.e. an enhanced release of corticosterone has been demonstrated in LAB mice [45]. Interestingly, we could not detect any polymorphisms in *Mt1*, which points to causes other than genetic variations on the expression level. Still, the effect of differential expression is inherited, as the measured differences seem to be consistent over generations and independent of gender.

The highlight of our transcriptome analysis, the lysosomal cysteine peptidase *Ctsb*, is an abundant and ubiquitously expressed member of the papain family C1, and contributes to the terminal degradation of proteins in the lysosome. However, there is increasing evidence that *Ctsb* has more specific functions in health and disease. In particular, KO mice have demonstrated that *Ctsb* plays a major role in pathological trypsinogen activation in the early course of experimental pancreatitis, and contributes to TNF-alpha induced hepatocyte apoptosis [46], [47].

Of the members of the C1 family of cysteine peptidases, *Ctsb* is by far the most abundant in the brain (compare http://www.brain-map.org/). However, *Ctsb* KO mice do not show any obvious, distinct neurological phenotype [21], which is potentially due to compensation or even rescue of the KO by other family members. This is supported by the finding that *Ctsb* and cathepsin L (*Ctsl*) double KO mice exhibit severe brain damage, with neuronal loss and brain atrophy, underscoring the important role of *Ctsb* in brain physiology [48], [49].

Our gene expression data, which show strongly increased expression of *Ctsb* in LAB compared to HAB animals, and the identification of many genetic variations in *Ctsb* between our lines, make it an interesting candidate for further research into its role in the expression of emotional behavior, which (to our knowledge) has never been addressed in *Ctsb* KO mice before.

The polymorphisms we identified do not affect protein structure, but could definitely affect either mRNA stability [50], or the recruitability of transcription enhancing or repressing factors [16], [51], [52]. Here, we demonstrated a pronounced effect of *Ctsb* deficiency on anxiety- and depression-like behaviors in mice. Although our major findings are restricted to female mice, we could also find moderate signs of increased anxiety and decreased explorative drive in male *Ctsb*-deficient animals. Grooming frequency on the EPF was higher in the HET group compared to both KO and WT mice, pointing to more displacement activity, i.e. arousal. Similarly, the frequency of explorative head-dips was lower in these animals, indicating that the effects of *Ctsb* deficiency can also be observed in male mice. As already supported by other KO studies [53], this modest effect could be compensated for by other factors (e.g., other cathepsins) in the complete KO mice. Compared to male mice, females showed pronounced differences on numerous measurements in the OF test and FST. These clearly underline *Ctsb* deficiency as a cause of increased anxiety-related and depression-like behaviors [46]. Similar results, regarding the relationship of *Ctsb* with changes in the brain, have so far only been shown in connection with the urokinase-type plasminogen activator receptor that participates in the functioning, development and disorders of the speech cortex, with a link to epilepsy [54], [55]. Another hint for the possible brain function of *Ctsb* is that its murine and human variants have similar biochemical properties, and participate in the production of β-amyloid peptides. Thus, lower levels of *Ctsb* could have both neurotoxic and, under certain conditions, neuroprotective effects by causing a decrease in the amounts of β-amyloid peptides [56], [57], [58].

There are many possible reasons for detecting these pronounced differences in female mice only. First of all, the different hormonal constitution might lead to a more distinct effect of *Ctsb* deficiency in males and females. Compensatory action of other gene products might also be more or less effective in different genders, a view which is supported by evidence that these hormones regulate cathepsins [59]. Sexual dimorphism also affects the normal concentrations of corticosterone in mice and rats and cortisol in humans, respectively. Nearly twice the concentration of corticosterone can be observed in females compared to males [60], which could imply a dimorphism caused by different sensitization of receptor mechanisms. Other prominent sexual dimorphisms include the actions of estrogen and testosterone, and the different effects of neuroimmunoendocrine processes [61]. These same mechanisms might account for the differences we observe in prevalence rates for both anxiety and depressive disorders. Most clinical studies dealing with these disorders show that women seem to be affected by them almost twice as frequently as men [62], [63], [64], so differently pronounced action of single candidate genes on specific endophenotypes could well be sex-specific.

Thus, in summary, we demonstrate the functional importance of *Ctsb* in anxiety-related and depression-like behaviors using KO mice. Our findings suggest that *Ctsb*, as well as the other genes we identified as differentially expressed in HAB *vs.* LAB mice, are likely to represent genetic underpinnings of anxiety- and depression-related behaviors. Although our findings in KO *vs.* HET and WT mice were restricted to female mice, they might mirror sexual dimorphisms, and the multifactorial nature of evolutionary old and well-conserved mechanisms regulating anxiety- and depression-like phenotypes.

## Materials and Methods

### Animals and behavioral tests

All animals were kept in the animal facility of the Max Planck Institute of Psychiatry, under standard housing conditions (room temperature 23±2°C, relative air humidity 60±5%, 12 h/12 h dark/light cycle with lights on at 7 am). Animals were kept in groups of two to four per cage, with light levels not exceeding 100 Lux at any time. Mice from each line were selected from generations G16-32, based on their anxiety-related behavior, as measured by their performance on the EPM. The animals were also tested with the TST [15] for 6 minutes to assess their depression-like coping strategy. Through heterozygous breeding, we created litters of mice containing both *Ctsb* deficient animals and control (non-deficient) animals (sequences of genotyping primers are available upon request). 42 male and 55 female mice *Ctsb* KO, HET and their WT littermates [46], [65] were tested for locomotion and anxiety-related behavior in the EPM and OF tests for 5 minutes, for depression-like behavior in the FST and TST for 6 minutes each [15], and for stress hormone response to a 15 minute SRT as described by Touma *et al.* [60]. The OF test we used had illumination of 10 Lux in the central part of the field. To assess explorative behavior, an EPF test [66] was performed for 5 minutes. The apparatus we used for this test consisted of a centered platform of grey polyvinylchloride 40 cm high and 10 cm in diameter, mounted to a wooden plate (42×42 cm) and surrounded by three wooden walls (w×h: 42×58 cm), and left open at the front for observation. The metrics we assessed on the EPF test were the frequency and duration of rearings, the delay before the first rearing, and the frequency of exploratory head-dips over the platform edge and the frequency of grooming behavior, which are indicative of displacement activity.

Testing was started on the *Ctsb* KO mice and their littermates at the age of eight weeks, starting with the EPM test on day 1, and continuing to TST on day 3, OF on day 7, EPF on day 9, SRT on day 11, and FST on day 14. All of the behavioral tests were digitally recorded or video-taped. The results of the EPM test, FST and TST were analyzed as in previous studies [15], [16]. The same evaluation scheme was applied to the EPF test, by counting the frequency of head-dips when mice reached the platform's edge with their shoulder line, the frequency, latency and duration of rearing, and the frequency of grooming behavior. The OF tests were analyzed using Anymaze v. 4.71 (Stoelting&Co., Wood Dale, IL). The order in which the mice were tested was random, and evaluation was performed by a trained observer who was not aware of the lines of the mice. All chemicals and reagents not declared otherwise were purchased from Sigma-Aldrich (Taufkirchen, Germany).

### Ethics statement

The government of Upper Bavaria (Oberbayern) approved our animal experiments (approvals IDs – AZ: 55.2-1-54-2531-64-07 and 55.2-1-54-2531-73-02), which were conducted according to the current regulations for animal experimentation in Germany and the European Union (European Communities Council Directive 86/609/EEC).

### Tissue dissection by micropunching

Animals were anesthetized and killed by decapitation at the age of ten weeks, three days after the TST. For MPIP24k gene expression analysis, six male HAB and LAB mice from breeding generation 16 were used, while for subsequent qPCR analysis, we used eight female HAB, LAB and CD1 mice from generation 22. A further group of eight male mice from each line, from generation 29, was used for micropuncture. The brains of the mice were collected, dissected into 200 µm slices, and mounted from rostral to caudal to Superfrost microscope slides (Menzel, Braunschweig, Germany) in a cryostat (Microm MH50, Microm, Walldorf, Germany). The brain areas of interest were acquired from these slices by micropuncture, through a method described in previous studies [67], utilizing punchers with a diameter of 0.5 and 1 mm (Fine Science Tools, Heidelberg, Germany). The brain regions collected included the Cg, the NAc core and shell, the PVN, the SON, and the BLA, CeA and medial amygdala (MeA). Punches of 1 mm diameter were collected from bregma +1.3 mm to +0.9 mm, twice sampling the tissue medially about 0.5 mm from the dorsal tissue border to receive the Cg, and bilaterally sampling the NAc core, which inevitably included a minor part of its shell around the anterior commissure. Further tissue was collected medially 0.8 mm above the ventral tissue limit (Ø = 1 mm), and bilateral-dorsolaterally from the optic tract (Ø = 0.5 mm), to acquire tissue from the PVN and SON, from bregma −0.56 mm to −0.96 mm. Amygdala tissue samples were collected bilaterally from two slides for each region with 1 mm diameter punches. CeA was collected from bregma −0.96 mm to −1.36 mm dorsomedially from the ventral end of the external capsule, MeA from −1.16 mm to −1.56 mm dorsolaterally from the optic tract, and BLA/LA was collected from bregma −1.36 mm to −1.76 mm from in between the bifurcation of the external capsule. All coordinates were based on the Mouse Brain Atlas [68].

### Tissue laser microdissection

Six male HAB, six male NAB, and six male LAB animals from generation 25 were sacrificed using the previous procedure, except for obtaining 25 µm slices in the cryostat (Microm). Only brain slices containing the brain areas of interest were sampled, including the anterior part of the Cg, the PVN, the SON, the anterior DG, the CeA, and the BLA. For the DG, the same coordinates as for the PVN and SON were used. To cover the whole area of interest, an overall depth of 400 µm per region was chosen. After sampling the Cg, BLA and CeA brain regions, four brain slices were mounted onto an LMD6000 metallic frame slide, which was covered with a membrane of polyethylene terephthalate (Leica Microsystems Deutschland, Bensheim, Germany), while the following four slices were mounted onto Superfrost slides (Menzel). This procedure was repeated for the next eight slices. For the region containing the PVN, SON and DG, only the first two slices were mounted to the LMD6000 frame, with the next two mounted to the Superfrost slides, and so on, again alternating between them. Only the LMD6000 slides were used for further processing, and these slides were stored at −80°C. Before we performed laser-microdissection, the brain slices on the LMD6000 frame slides were stained with cresyl violet. Our staining protocol started with staining the slides for 90 seconds in cresyl violet, followed by washing them in 70% and 96% ethanol for 20 seconds each, and finally washing them in isopropanol for 5 minutes. The slides were refrozen after staining, and were then processed with the laser-microdissection microscope (AS LMD, Leica). For dissecting these brain areas, we chose a magnification of 100×, laser power between 80–100%, and speed varying between 1 and 4. Cut-out brain areas were stored in the caps of 0.2 ml PCR soft tubes (Biozym Scientific, Hessisch Oldendorf, Germany), and were cooled with dry ice immediately after the completion of one brain region.

### Total RNA isolation and amplification

Total RNA was extracted in presterilized 1.5 ml safelock tubes (Eppendorf, Hamburg, Germany), using a standard TRIzol (Invitrogen, Karlsruhe, Germany) chloroform protocol. After the sample tissue was homogenized by adding 300 µl TRIzol with a pipette, 1 µl linear acrylamide (5 mg/ml, Ambion, Austin, TX) and 60 µl chloroform (Carl Roth, Karlsruhe, Germany) were added, and the samples were vortexed. This was followed by centrifugation for 5 min at 18°C and 13,000 rpm. The RNA was then precipitated by applying 180 µl isopropanol (Carl Roth) overnight at −20°C, centrifuging the sample at 4°C and 13,000 rpm for 30 min, and then washing it twice in 500 µl 70% ethanol (Carl Roth), with ten-minute centrifugation steps at 4°C and 13,000 rpm in between. After the last centrifugation step, all remaining liquid was removed with a pipette, and the pellets were dried in an incubator for 15 min at 45°C and stored solved in 13 µl of water (Ampuwa, Braun Melsungen, Melsungen, Germany).

For the MPIP24k experiment, the total RNA we extracted was amplified and dye-coupled in two rounds, using Ambion's Amino Allyl MessageAmp aRNA kit (Ambion). T7 oligo(dT) primers were used for the first round of reverse transcription to select specifically for mRNA, and random hexamer primers were used for the second round of reverse transcription.

For the Illumina MouseWG-6 experiment, the RNA was amplified using the Illumina TotalPrep RNA Amplification kit (Ambion). 5 µg of material per sample was required for loading the microarray slides. We ensured correct quantification by measuring optic density in a NanoPhotometer (Implen, Munich, Germany), and through additional analysis on agarose gel. Samples not fulfilling all of our homogeneity criteria (samples with inadequate concentrations or size distribution) were excluded from further analysis.

### Hybridization and quantification of the MPIP24k arrays

Ten array slides per brain region (MPIP24k arrays containing 24,192 probes, MPI of Psychiatry, Munich, Germany [69], [70], platform available in NCBI/GEO with accession number: GPL7467; http://www.ncbi.nlm.nih.gov/geo/query/acc.cgi?acc=GPL7467) – serving as technical replicates - were prehybridized prior to the experiment in a prehybridization buffer, for 1 h at 42°C. The buffer consisted of 125 ml formamide, 62.5 ml 20×SSC, 2.5 ml 10% SDS, 2.5 ml BSA at 10 mg/ml and 57.5 ml of water. The array slides were then washed in water and isopropanol, and dried by three minutes of centrifugation (Megafuge 1.0R, Heraeus, Hanau, Germany) in 50 ml tubes at 1500 g (Sarstedt). Dye-coupled aRNA samples were mixed with the opposite dye-coupled samples of the relevant regions from the other mouse line, and were loaded with a hybridization buffer (consisting of 500 µl formamide, 250 µl 20×SSC, 10 µl 10% SDS, 5 µl mouse-COT1-DNA at 20 mg/ml (Invitrogen) and 40 µl poly adenylic acid at 2.5 µg/µl (Amersham Biosciences) to five arrays each under m-Series LifterSlips (Menzel). All arrays were hybridized at 50°C for 16–17 h in separate hybridization chambers. The arrays were then washed in 2×SSC and 0.1% SDS for 5 minutes at 42°C, for 10 min at room temperature in 0.1×SSC and 0.1% SDS, four times in 0.1×SSC for 1 min each, and finally for 20 s in 0.01×SSC solutions. The slides were dried by three minutes of centrifugation at 1,500 rpm. Then, the arrays were scanned on a PerkinElmer ScanArray 4000 (PerkinElmer Life and Analytical Sciences, Shelton, CT) laser scanner, using automatic focusing and laser power between 60 and 80 for Cy3, and 40 and 70 for Cy5. This ensured that, on average, the same fluorescence intensities were reached in both dyes, and that not more than 1–2% of spots had fluorescence intensities above the saturation point. Quantification of all array data was performed with QuantArray software (GSI Lumonics, Billerica, MA), by applying a fixed-circle quantification protocol, with manual positioning of all grids over the hybridized spots. To provide a negative control for the hybridization and evaluation procedure, excess aRNA was used for an additional hybridization in both Cy3 and Cy5 combinations.

### Hybridization and quantification of the Illumina MouseWG-6 arrays

The platform description is available at NCBI GEO, accession Number GPL4234 (http://www.ncbi.nlm.nih.gov/geo/query/acc.cgi?token=fjqlzekgyecycng&acc=GPL4234). Each microarray slide had the capacity for six samples. Samples containing the same brain regions were hybridized in the same batch, so a maximum of six individual mice of each breeding line were compared per batch. Reagents and material for the analysis were provided by Illumina. In brief, each sample was mixed with hybridization buffer and loaded onto the designated array field, and the slides were then put into hybridization chambers and incubated for 17 h in Illumina incubation chambers. The arrays were washed in several steps, incubated with Cy3-Streptavidin, again washed several times, and dried by centrifugation. Finally, data on fluorescence was gathered using a BeadStation scanner (Illumina), and analyzed with the BeadStudio (Illumina) software. The manufacturer's built-in controls were also analyzed, including hybridization controls and sample dependent parameters. Only microarrays meeting Illumina's recommended quality control criteria were used for further evaluation.

### Quantitative PCR (qPCR)

Candidate genes were selected for further analysis if they showed at least 40% greater regulation in one region, and at least 30% greater regulation in all other regions, in HAB mice relative to LAB mice, with adjusted p-value<0.05.

Three 200 µm coronal sections were also taken at different brain levels, for representative whole brain analyses. A maximum of 1 µg of total RNA was reverse transcribed with Superscript II (Invitrogen, Karlsruhe, Germany) after DNAse treatment. For quality control, a small aliquot of each cDNA was analyzed on an agarose gel. cDNA of male or female HAB or LAB mice was analyzed by qPCR, using the QuantiFast SYBR Green PCR Kit (Qiagen, Hilden, Germany). The oligonucleotide primers were designed based on the Primer3 algorithm [71], and were then purchased from Sigma-Aldrich. We selected gene products for quantification by qPCR if they showed: significant expression differences between HAB and LAB mice with an adjusted p-value of <0.10, at least 30% greater regulation between lines, and differential expression in the microarray experiment in all, or at least three, analyzed regions. All the primers we used for qPCR are listed in Table S1. Experiments were performed in duplicates on the Lightcycler®2.0 instrument (Roche Diagnostics, Mannheim, Germany) using the following PCR process: initial denaturation at 95°C for 10 min, followed by 40 cycles of denaturation (95°C for 10 s), and then a combined annealing and extension phase (60°C for 30 s). At the end of every run, a melting curve (50–95°C with 0.1°C/s) was used to ensure the quality of the PCR product. Crossing points (Cp) were calculated with LightCycler® 4.0 software (Roche Diagnostics), using the absolute quantification fit points method. The threshold and noise band were set to the same level in all compared runs. Relative gene expression was determined by the 2^−ΔΔCT^ method [72]. Cp were normalized to the housekeeping genes *Gapdh*, *Hprt1*, *Atp2b1*, *Rpl13a* and *Polr2b*, or any combination of two of the mentioned genes. Fold regulation values were calculated relative to the expression mean of the group displaying the lowest expression.

### *In situ* hybridization (ISH)

ISH using ^35^S-UTP labeled ribonucleotide probes was performed as described in previous studies [73], [74] to detect *Syt4* mRNA. Briefly, sets of sections for each riboprobe ISH were fixed in 4% paraformaldehyde, and acetylated in 0.25% acetic anhydride in 0.1 M triethanolamine/HCl. Afterwards, the slides were dehydrated with progressively higher ethanol concentrations, degreased with chloroform, rinsed in ethanol and then air dried. The antisense cRNA probe for *Syt4* (470 bp) was transcribed from a linearized plasmid, and labeled using SP6 polymerase transcription systems, using a standard labeling reaction mixture consisting of 1.5 µg of linearized plasmid, 1× transcription buffer, 0.12mCi of ^35^S-UTP, 1 mM dNTPs, 16.7 mM DTT, 40 U of RNAse inhibitor, and 20 U of the polymerase. The reaction mix was incubated at 37°C for three hours, and the labeled probe was then separated from free nucleotides with spin columns (Qiagen, [74]). The tissue sections (5 sections per slide) were saturated with 100 µl of hybridization buffer (Tris HCl, EDTA, NaCl, formamide, 5 M DTT, Dehnhard's solution, DEPC H_2_0, 50% dextran sulfate) containing 106 cpm ^35^S labeled riboprobe. Brain sections were coverslipped and incubated in humid chambers for 18–22 h, at a temperature of 55°C. After this was done, the slides were rinsed in 2×SSC, treated with RNAse A (20 mg/l), washed in progressively less concentrated SSC solutions, dehydrated by progressively more concentrated ethanol solutions, and air-dried, before they were photographed with Kodak BioMax MR films (Amersham, Braunschweig, Germany) over a 14-day period. Afterwards, the films were fixed, developed, and digitized, and the radiation-induced blackening of different brain regions was quantified by image analysis, using Scion Image (Version 4.0.3.2, Scion Corporation, Frederick, MD) software. Autoradiograms were analyzed by computer assisted optical density readings (relative grey intensity as a measure of relative expression) of the respective area, as well as the relative size of the labeled area. Three to six brain sections of each individual were quantified by an observer unaware of the individual's breeding line, and the highest expression (hybridization signal of a certain region, minus the background signal of a nearby structure that does not express the gene of interest) was used to calculate each mRNA expression, respectively.

### Sequencing of candidate genes

To identify polymorphic sites between the HAB and LAB mice, the *Ctsb* and metallothionein 1 (*Mt1*) coding genes were sequenced, as these were the two candidate genes whose involvement in diverse molecular pathways was best described. In both cases, about 2,500 bp of the gene promoter, all exons and introns (if the latter did not exceed 2,000 bp), and about 2,000 bp of the downstream enhancer regions (DER) were sequenced. Sequencing primers were designed to cover 500 to 600 bp.

Sequencing was also used to verify qPCR reaction products, and their primer binding sites. In this case, the fragments used were between 200 and 500 bp.

DNA was isolated from tail tips using the NucleoSpin Tissue (Macherey-Nagel) kit, from fresh fecal samples as described by Murgatroyd *et al.* [75], or sequencing was prepared based on cDNA. PCR reactions were set up in three HAB and three LAB samples. The primers we used are summarized in Table S4. All amplifications were carried out using *Taq* polymerase (Fermentas, St. Leon-Rot, Germany) in 25-µl reactions with the following procedure: initial denaturation at 94°C for 4 mins, 40 cycles of denaturation (94°C for 1 min each), annealing (52–66°C for 1 min) and extension (72°C for 1 min), followed by 10 min of final extension at 72°C. 15–20 µl of PCR or qPCR product were purified by washing the sample twice with nuclease-free water, using the NucleoFast 96 PCR Clean-up kit (Macherey-Nagel). Clean-up plates were centrifuged at 9°C and 4,500 g for 10 min (Heraeus Multifuge 4KR, Thermo Fisher Scientific, Waltham, MA), and resolved for ten more minutes in 25 µl of water on a shaker (Eppendorf). 2.4 µl of cleaned up PCR product was used for the sequencing reaction (BigDye Terminator Kit, Applied Biosystems, Foster City, CA), with 1.2 µl of sequencing buffer, 0.4 µl BigDye reagent and 1 µl of the forward primer per sample. If sequencing reaction results were unclear or unreadable, the sequencing reaction was also performed with the reverse primer. Sequencing reactions were set up on ThermoFast 96 PCR plates (ABgene, Hamburg, Germany) using a PTC-225 Gradient MultiCycler (MJ Research, Miami, FL) using the following procedure: initial denaturation at 96°C for 1 min, 35 cycles of denaturation (96°C for 10 s), annealing (50°C for 5 s), and finally extension (60°C for 4 min). Sequencing reaction products were purified by washing them twice in 20 µl injection solution, using Montage SEQ96 plates (Millipore, Billerica, MA) on a vacuum pump (Biomek 2000 Laboratory Automation Workstation, Beckman Coulter, Fullerton, CA), and the reaction products were then transferred to 96 well plates. Sequences were determined by capillary electrophoresis on a 3730 DNA Analyzer (Applied Biosystems) at the HelmholtzZentrum's Institute of Human Genetics (Neuherberg, Germany). Sequence analysis and comparison were done using FinchTV Ver. 1.2 (Geospiza, Seattle, WA) and BioEdit Ver. 7.0.2 (Tom Hall, Ibis Biosciences, Carlsbad, CA) softwares.

### Statistical analysis

All data, except for data from high throughput gene expression profiling, was analyzed using SPSS Ver. 16.0.1 (Chicago, IL). The Kruskal-Wallis test (KWH) was used for comparisons over more than two groups, with subsequent Mann-Whitney tests (MWU), as well as sequential Bonferroni correction for multiple testing (if applicable).

For statistical evaluation of the MPIP24k arrays, analytic methods were applied as described in previous studies [76], [77]. In brief, an MA-plot was first generated to display the raw fluorescence intensities of Cy5 (R) and Cy3 (G), with *M = log_2_ R/G* and *A = log_2_* 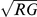. The data were then normalized to exclude systematic and technical errors. The data were first normalized twice, by subtracting a function *c* from the logarithms of the fluorescence intensities (*log_2_ R* and *log_2_ G*). First, a global normalization was performed, based on the assumption that R and G correlate:
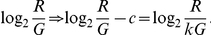
In the second normalization step, an intensity-dependent normalization was added, which was done by applying a LOESS smooth operation, as described in the R software package (http://www.r-project.org). This was calculated with:
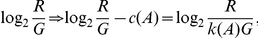
where c(A) stands for the LOESS smooth of the MA-plot. 40% of the values in the MA-plot were used to calculate the LOESS smooth. In several subsequent normalization steps, we minimized differences that resulted from an unequal distribution of probes in the array production, or from unbalanced fluorescence intensities within one array slide. All data were then merged into a matrix. P-values for multiple testing were calculated by permutation, and are therefore called adjusted p-values.

For the Illumina Mouse WG arrays, Illumina BeadStudio gene expression results were analyzed using a statistical procedure similar to the one for the MPIP24k experiment. All analyses were performed using R-packages, based on ‘beadarray’ described by Dunning *et al.* [78], which simplifies comparisons between high numbers of arrays. First, pair-wise box plots were generated to compare the mean amount of expression within each line and brain region. Normalization for expression values was applied to all samples with the ‘QSpline’ function. Clustering using the ‘hclust’ function shows that each brain region per line shows similar expression patterns. Three samples from different brain regions were identified as inadequate during the scan process, and have therefore been excluded from further analysis. For differential expression analysis, the functions of the ‘limma’ package were applied to *log_2_*-transformed values. The resulting matrix has been used for all subsequent analyses. Significantly regulated genes were ranked using an empirical BAYES method, as implemented in the limma R-package [79], [80].

## Supporting Information

Table S1 List of primers for qPCR with chromosomes and exons the primers hybridized to. List is sorted alphabetically according to the gene symbols. Candidate genes from the gene expression microarray experiment are marked by asterisk after the gene symbols, all others were used as housekeeping genes.
(DOC)

Table S2 Variations identified in or around the amplified fragments used in qPCR. Variation type refers to single nucleotide polymorphisms (SNPs), deletions or insertions, the genomic position to the physical position on the respective chromosome (Mouse Genome Build 37), HAB and LAB to their line-specific allele, location in the gene to the functional structure of the variation locus, relative (rel.) position to the gene locus, position in mRNA to the spliced mRNA and SNP identifier to already described polymorphisms.
(DOC)

Table S3 Variations identified in the cathepsin B *(Ctsb)* gene. Variation type refers to single nucleotide polymorphisms (SNPs), deletions or insertions, the genomic position to the physical position on chromosome 14 (Mouse Genome Build 37), HAB and LAB to their line specific allele, location in the gene to the functional structure of the variation locus (downstream enhancer region: DER), relative (rel.) position to the *Ctsb* locus, position in mRNA to the spliced mRNA and SNP identifier to already described polymorphisms.
(DOC)

Table S4 Primer sequences used for sequencing of the cathepsin B (*Ctsb*), metallothionein 1 (*Mt1*) genes as well as for the fragments analyzed by qPCR including the PCR fragment length resulting from each reaction.
(DOC)
