# Supplementary material for: Profiling Trait Anxiety: Transcriptome Analysis Reveals Cathepsin B (Ctsb) as a Novel Candidate Gene for Emotionality in Mice
Source: PLoS One. 2011 Aug 29;6(8):e23604. doi: 10.1371/journal.pone.0023604 (PMC3163650; doi:10.1371/journal.pone.0023604)
Supplement: Table S1 — List of primers for qPCR with chromosomes and exons the primers hybridized to. List is sorted alphabetically according to the gene symbols. Candidate genes from the gene expression microarray experiment are marked by asterisk after the gene symbols, all others were used as housekeeping genes. (DOC) [file pone.0023604.s001.doc]

**Table S1**

| **Chr.** | **Gene symbol** | **Orient-ation** | **Exon** | **Primer sequence  5'🡪3'** | **Prod. Size [bp]** |
| --- | --- | --- | --- | --- | --- |
| 1 | *5230400* | forward |  | TAT GAA ATG GAA TAC ACC GAA GG | 210 |
|  | *G24Rik** | reverse |  | ATC TGC TGG TCT TGA AAA TGA AA | |
| 2 | *Abca2** | forward | 47 | CAT CAG CTT CGA GGA AGA GC | 206 |
|  |  | reverse | 48 | CAT TCG GGG AGG ATG GTA G | |
| 11 | *Aldh3a2** | forward | 9 | TCC TGC TGA AGC AGT TCA AC | 157 |
|  |  | reverse | 10 | ACA GGG AAG TCC ACC AGA TC | |
| 7 | *Apbb1** | forward | 13 | TTC TCT CCT TCC TGG CTG TG | 162 |
|  |  | reverse | 14 | GAC ACT TCT GGT AGC GGA GC | |
| 10 | *Atp2b1* | forward | 1 | CAT TAC GGA AAA TAC AGG AGA GC | 182 |
|  |  | reverse | 2 | TGC TTC CCA GAC TAA CTG AAG AA | |
| 11 | *Ccdc104** | forward | 1 | AAG AAG AGG ACG AAG TGG AAT G | 180 |
|  |  | reverse | 2 | TCT TGA TGG ATT TCT GTG TAG G | |
| 16 | *Coro7** | forward | 1 | AGG TGT CCA AGT TTC GGC ATA | 113 |
|  |  | reverse | 2 | GCA GCT TGA TTT GAT GTG GTT | |
| 14 | *Ctsb** | forward | 1 | CTG CGC GGG TAC TTA GGA GT | 148 |
|  |  | reverse | 2 | CAG GCA AGA AAG AAG GAT CAA G | |
| 14 | *Dgkh** | forward | 27 | GGA GCC TCC TCT GGA TTG TA | 211 |
|  |  | reverse | 29 | AGT GTG ACC ACC CCT CAG AC | |
| 5 | *Dgkq** | forward | 22 | TAC AAG GTG GGC TAC GCT CT | 188 |
|  |  | reverse | 23 | CAA GGT GTC CAC TCG GGT AT | |
| 17 | *Enpp5** | forward | 3 | TGC CCA TCC TAA TCT AAC GG | 186 |
|  |  | reverse | 4 | GGA TGC ATT TCT GCT AAT GC | |
| 5 | *Ep400** | forward | 1 | GGT GCA AGC GAA CGG GAT AG | 202 |
|  |  | reverse | 2 | GAC TGG CTG ATG GAG CGA AAG | |
| 6 | *Gapdh* | forward | 3 | CCA TCA CCA TCT TCC AGG AGC GAG | 227 |
|  |  | reverse | 4.5 | GAT GGC ATG GAC TGT GGT CAT GAG | |
| 3 | *Gig1** | forward | 8 | CAG GAG AGC AGA GAC AGC AC | 185 |
|  |  | reverse | 9 | AAT GGC TCA GTC AAT GAA CC | |
| 19 | *Gnaq** | forward | 6 | CAT GGA GGA GAG CAA AGC AC | 178 |
|  |  | reverse | 7 | GGT TCA GGT CCA CGA ACA TT | |
| 7 | *Hbb-b1** | forward | 1 | CCT GTG GGG AAA GGT GAA C | 148 |
|  |  | reverse | 2 | GGC CTT CAC TTT GGC ATT AC | |
| 9 | *Hmgn3** | forward | 5 | AGG TGC TAA GGG GAA GAA GG | 171 |
|  |  | reverse | 6 | GTC CCG AGA GGT ACG TGA AA | |
| X | *Hprt1* | forward | 8 | GTC AAG GGC ATA TCC AAC AAC AAA C | 240 |
|  |  | reverse | 3 | CCT GCT GGA TTA CAT TAA AGC ACT G | |
| 1 | *Kcnh1** | forward | 10 | ACG CCC TTC AGA AAG TGC TA | 182 |
|  |  | reverse | 11 | GTG GTC AGG AGG CAG GAT AA | |
| 3 | *Mbnl1** | forward | 1 | ACG ACC AGA CAC GGA ATG TAA | 168 |
|  |  | reverse | 2 | CGC CCA TTT ATC TCT AAC TGT GT | |
| 8 | *Mmp15** | forward | 1 | CCG AGA TGC AGA GTT TCT ATG G | 189 |
|  |  | reverse | 2 | TGA AGG TCA GGT GGT AAT TGT TC | |
| 8 | *Mt1** | forward | 1 | CTA AGC GTC ACC ACG ACT TC | 157 |
|  |  | reverse | 2 | GCA CTT GCA GTT CTT GCA G | |
| 6 | *Npy** | forward | 1 | GCT CTA TCT CTG CTC GTG TGT TT | 175 |
|  |  | reverse | 2 | GTG TCT CAG GGC TGG ATC TCT | |
| 14 | *Pdhb** | forward | 9 | TCG AAG CCA TAG AAG CCA GT | 173 |
|  |  | reverse | 10 | AGG CAT AGG GAC ATC AGC AC | |
| 5 | *Polr2b* | forward | 9 | CAA GAC AAG GAT CAT ATC TGA TGG | 157 |
|  |  | reverse | 7 | AGA GTT TAG ACG ACG CAG GTG | |
| 14 | *Pxk** | forward | 16 | AAC AGT GAG GAG CAG CCA GT | 161 |
|  |  | reverse | 19 | GGT AAT GCT GAA GAC AGT CC | |
| 7 | *Rab6** | forward | 8 | GCC TTT CTT GCC TCT TCC TTT | 244 |
|  |  | reverse | 8 | GCT CAT AGC CTG GAG CTG TC | |
| 7 | *Rpl13a* | forward | 8 | CAC TCT GGA GGA GAA ACG GAA GG | 181 |
|  |  | reverse | 10 | GCA GGC ATG AGG CAA ACA GTC | |
| 2 | *Slc1a2** | forward | 9 | ACC GAA TGC AGG AAG ACA TC | 221 |
|  |  | reverse | 10 | AAT TGG CTG AGA ATC GGG TC | |
| 15 | *Slc25a17** | forward | 1 | CAT GGC CTC TGT GCT GTC CTA C | 139 |
|  |  | reverse | 2 | GAA GCC GAA GTC TAG CAG TAT CCA | |
| 5 | *Smarcd3** | forward | 1 | CAA TAC TTT TAA CCC TGC GAA GC | 154 |
|  |  | reverse | 2 | GTC CAA CTC AAT GAC CAA ACT CT | |
| 19 | *Spnb3** | forward | 24 | GCC CAG ACC ATC AAA CAA CT | 221 |
|  |  | reverse | 25 | TCA CGC TCC TGT ATC CAC TG | |
| 18 | *Syt4** | forward | 2 | TGA TGT CAT TGG AGA AGT CCT G | 168 |
|  |  | reverse | 3 | ACC ACA GTG AGC GTG TTT GT | |
| 19 | *Stx3 ** | forward | 10 | CTT CTA CCA TTG GGG GCA TA | 145 |
|  |  | reverse | 11 | TGC CCT GTG TTG TGA GTT TC | |
| 3 | *Tpd52* | forward | 3 | AGT ATT GGC CGC AAA AGA GA | 194 |
|  |  | reverse | 4 | CTG AGC CAA CCG ATG AAA AT | |
| 12 | *Trib2** | forward | 1 | TCG GGA AAT ACT TAC TGT TGG AG | 219 |
|  |  | reverse | 2 | AGC TTC GCT CAA AGA ACA CAT AG | |
| 17 | *Ttbk1** | forward | 13 | ATC AGT GTG TCC ATG CCT GT | 148 |
|  |  | reverse | 14 | ACT GTT TGG GAC GGA GGT C | |
| 18 | *Ttr** | forward | 1 | CCT CGC TGG ACT GGT ATT TG | 121 |
|  |  | reverse | 2 | TTA CAG CCA CGT CTA CAG CAG | |
| 11 | *Zfp672** | forward | 1 | GTC CTC AAG GTC ACA CAA TTA GTC | 207 |
|  |  | reverse | 2 | CAG ACA TGA GTG TAG GGT GCA AG | |
