# Supplementary material for: Profiling Trait Anxiety: Transcriptome Analysis Reveals Cathepsin B (Ctsb) as a Novel Candidate Gene for Emotionality in Mice
Source: PLoS One. 2011 Aug 29;6(8):e23604. doi: 10.1371/journal.pone.0023604 (PMC3163650; doi:10.1371/journal.pone.0023604)
Supplement: Table S2 — Variations identified in or around the amplified fragments used in qPCR. Variation type refers to single nucleotide polymorphisms (SNPs), deletions or insertions, the genomic position to the physical position on the respective chromosome (Mouse Genome Build 37), HAB and LAB to their line-specific allele, location in the gene to the functional structure of the variation locus, relative (rel.) position to the gene locus, position in mRNA to the spliced mRNA and SNP identifier to already described polymorphisms. (DOC) [file pone.0023604.s002.doc]

**Table S2:**

| **Gene** | **Variation type** | **Genomic position** | **HAB** | **LAB** | **Location in the gene** | **Rel. position** | **Pos. in mRNA** | **SNP identifier** |
| --- | --- | --- | --- | --- | --- | --- | --- | --- |
| *Hmgn3* | SNP | 83,005,853 | A | G | Exon 5 | 34,440 | 524 | rs13474367 |
| *Hmgn3* | SNP | 83,004,015 | C | T | Exon 6 | 36,278 | 1,249 | rs13474366 |
| *Pdhb* | SNP | 8,998,797 | T | G | Exon 10 | 6,666 | 1,148 |  |
| *Stx3* | SNP | 11,851,695 | T | A | Intron 13 | 42,199 |  | rs36877510 |
| *Stx3* | SNP | 11,850,873 | C | A | Exon 15 | 43,021 | 1,757 | rs30797387 |
| *Stx3* | SNP | 11,850,865 | C | G | Exon 15 | 43,029 | 1,765 | rs31307540 |
| *Stx3* | SNP | 11,850,852 | G | A | Exon 15 | 43,042 | 1,778 | rs30854920 |
| *Stx3* | SNP | 11,850,826 | G | A | Exon 15 | 43,068 | 1,804 | rs30499364 |
