# Supplementary material for: Profiling Trait Anxiety: Transcriptome Analysis Reveals Cathepsin B (Ctsb) as a Novel Candidate Gene for Emotionality in Mice
Source: PLoS One. 2011 Aug 29;6(8):e23604. doi: 10.1371/journal.pone.0023604 (PMC3163650; doi:10.1371/journal.pone.0023604)
Supplement: Table S3 — Variations identified in the cathepsin B (Ctsb) gene. Variation type refers to single nucleotide polymorphisms (SNPs), deletions or insertions, the genomic position to the physical position on chromosome 14 (Mouse Genome Build 37), HAB and LAB to their line specific allele, location in the gene to the functional structure of the variation locus (downstream enhancer region: DER), relative (rel.) position to the Ctsb locus, position in mRNA to the spliced mRNA and SNP identifier to already described polymorphisms. (DOC) [file pone.0023604.s003.doc]

**Table S3:**.

| **Variation type** | **Genomic position** | **HAB** | **LAB** | **Location in the gene** | **Rel. position** | **Pos. in mRNA** | **SNP identifier** |
| --- | --- | --- | --- | --- | --- | --- | --- |
| SNP | 63,739,084 | C | T | Promoter | -2,269 |  |  |
| SNP | 63,739,124 | C | G | Promoter | -2,229 |  |  |
| Insertion | 63,739,210 | GAGA | - | Promoter | -2,143 |  |  |
| SNP | 63,739,257 | C | T | Promoter | -2,096 |  |  |
| SNP | 63,739,273 | C | T | Promoter | -2,080 |  |  |
| SNP | 63,739,308 | G | A | Promoter | -2,045 |  |  |
| Insertion | 63,739,394 | C | - | Promoter | -1,959 |  |  |
| SNP | 63,739,609 | G | A | Promoter | -1,744 |  |  |
| SNP | 63,740,071 | G | C | Promoter | -1,282 |  |  |
| SNP | 63,740,540 | C | A | Promoter | -826 |  | rs30963834 |
| SNP | 63,740,968 | T | C | Promoter | -398 |  | rs30962992 |
| SNP | 63,741,271 | G | T | Promoter | -95 |  |  |
| SNP | 63,741,362 | C | T | Exon 1 | 10 | 10 | rs30962990 |
| SNP | 63,741,423 | A | G | Intron1 | 71 |  | rs30962988 |
| SNP | 63,752,252 | A | G | Exon 2 | 10,900 | 150 |  |
| SNP | 63,752,362 | T | C | Intron 2 | 11,010 |  |  |
| SNP | 63,752,540 | A | G | Intron 2 | 11,188 |  |  |
| SNP | 63,752,557 | C | T | Intron 2 | 11,205 |  | rs30973898 |
| SNP | 63,752,683 | C | T | Intron 2 | 11,331 |  | rs16791841 |
| SNP | 63,752,876 | G | A | Exon 3 | 11,524 | 276 | rs16791842 |
| SNP | 63,753,119 | C | T | Intron 3 | 11,767 |  | rs16791844 |
| SNP | 63,753,163 | A | G | Intron 3 | 11,811 |  |  |
| SNP | 63,753,224 | A | G | Intron 3 | 11,872 |  | rs30972751 |
| SNP | 63,753,468 | T | C | Intron 3 | 12,116 |  |  |
| SNP | 63,753,529 | C | T | Intron 3 | 12,177 |  |  |
| SNP | 63,753,684 | T | A | Intron 3 | 12,332 |  |  |
| SNP | 63,753,699 | C | G | Intron 3 | 12,347 |  |  |
| SNP | 63,753,705 | C | T | Intron 3 | 12,353 |  |  |
| Insertion | 63,753,712 | A | - | Intron 3 | 12,360 |  |  |
| SNP | 63,753,733 | T | A | Intron 3 | 12,381 |  |  |
| SNP | 63,753,745 | C | T | Intron 3 | 12,393 |  |  |
| SNP | 63,753,778 | C | T | Intron 3 | 12,426 |  |  |
| Deletion | 63,753,885 | - | AATAAAT CTAAGAG AAGGATG AGTCACT | Intron 3 | 12,533 |  |  |
| SNP | 63,753,914 | G | A | Intron 3 | 12,562 |  |  |
| SNP | 63,753,930 | T | G | Intron 3 | 12,578 |  |  |
| Deletion | 63,753,945 | - | TAAAAATAA GCCTGAAG | Intron 3 | 12,593 |  |  |
| SNP | 63,754,102 | A | G | Intron 3 | 12,750 |  |  |
| SNP | 63,754,112 | C | A | Intron 3 | 12,760 |  |  |
| Deletion | 63,754,125 | - | GGAA | Intron 3 | 12,773 |  |  |
| Insertion | 63,754,163 | ACA | - | Intron 3 | 12,811 |  |  |
| SNP | 63,754,221 | T | C | Intron 3 | 12,869 |  |  |
| SNP | 63,754,227 | C | T | Intron 3 | 12,875 |  | rs30972744 |
| SNP | 63,754,228 | G | A | Intron 3 | 12,876 |  |  |
| SNP | 63,754,279 | C | T | Intron 3 | 12,927 |  | rs30971633 |
| SNP | 63,754,724 | G | A | Intron 4 | 13,372 |  |  |
| Deletion | 63,754,741 | - | G | Intron 4 | 13,389 |  |  |
| SNP | 63,754,763 | T | C | Intron 4 | 13,411 |  |  |
| SNP | 63,754,764 | G | C | Intron 4 | 13,412 |  |  |
| SNP | 63,754,866 | A | G | Intron 4 | 13,514 |  |  |
| SNP | 63,754,907 | A | C | Intron 4 | 13,555 |  |  |
| SNP | 63,754,931 | A | T | Intron 4 | 13,579 |  |  |
| SNP | 63,754,948 | C | T | Intron 4 | 13,596 |  | rs30971631 |
| SNP | 63,755,037 | C | T | Intron 4 | 13,685 |  |  |
| SNP | 63,755,050 | C | G | Intron 4 | 13,698 |  |  |
| SNP | 63,755,110 | C | G | Intron 4 | 13,758 |  |  |
| SNP | 63,755,122 | C | A | Intron 4 | 13,770 |  | rs30971628 |
| SNP | 63,755,186 | C | T | Intron 4 | 13,834 |  |  |
| SNP | 63,755,315 | A | G | Exon 5 | 13,963 | 474 | rs13462712 |
| SNP | 63,755,474 | G | A | Intron 5 | 14,122 |  |  |
| Deletion | 63,755,498 | - | T | Intron 5 | 14,146 |  |  |
| SNP | 63,755,500 | G | T | Intron 5 | 14,148 |  |  |
| Deletion | 63,756,855 | - | AA | Intron 5 | 15,503 |  |  |
| SNP | 63,757,503 | T | C | Intron 7 | 16,151 |  |  |
| SNP | 63,758,010 | A | G | Intron 8 | 16,658 |  |  |
| SNP | 63,760,454 | G | A | Intron 8 | 19,102 |  | rs30969648 |
| SNP | 63,760,495 | G | A | Intron 8 | 19,143 |  | rs30969646 |
| Insertion | 63,760,849 | CACATGG TTTTGTAG ACAGTTCC | - | Intron 9 | 19,497 |  |  |
| SNP | 63,761,222 | C | T | Exon 10 | 19,870 | 1,151 | rs13462709 |
| SNP | 63,761,241 | T | G | Exon 10 | 19,889 | 1,170 |  |
| SNP | 63,761,536 | C | G | Exon 10 | 20,184 | 1,466 |  |
| SNP | 63,761,844 | G | T | Exon 10 | 20,492 | 1,774 | rs13462707 |
| SNP | 63,762,117 | C | G | DER | +144 |  |  |
| SNP | 63,762,512 | T | C | DER | +539 |  |  |
| SNP | 63,762,597 | A | C | DER | +624 |  |  |
| SNP | 63,762,653 | A | G | DER | +680 |  | rs30967661 |
| SNP | 63,762,668 | A | C | DER | +695 |  |  |
| SNP | 63,762,783 | T | A | DER | +810 |  | rs30967657 |
| SNP | 63,763,038 | C | A | DER | +1,065 |  |  |
| SNP | 63,763,124 | C | T | DER | +1,151 |  | rs30967655 |
| SNP | 63,763,140 | C | T | DER | +1,167 |  | rs30966673 |
| Insertion | 63,763,172 | CA | - | DER | +1,199 |  |  |
| Deletion | 63,763,184 | - | TC | DER | +1,211 |  |  |
| SNP | 63,763,230 | A | C | DER | +1,257 |  |  |
| Deletion | 63,763,303 | - | TTA | DER | +1,330 |  |  |
| SNP | 63,763,356 | C | T | DER | +1,383 |  |  |
| Insertion | 63,763,385 | CC | - | DER | +1,412 |  |  |
| Deletion | 63,763,400 | - | GTTT | DER | +1,427 |  |  |
| SNP | 63,763,441 | C | G | DER | +1,468 |  |  |
| SNP | 63,763,452 | A | C | DER | +1,479 |  |  |
| SNP | 63,763,470 | A | G | DER | +1,497 |  |  |
| SNP | 63,763,474 | A | G | DER | +1,501 |  |  |
| Insertion | 63,763,662 | ATA | - | DER | +1,689 |  |  |
| SNP | 63,763,715 | A | G | DER | +1,742 |  |  |
