# Supplementary material for: Profiling Trait Anxiety: Transcriptome Analysis Reveals Cathepsin B (Ctsb) as a Novel Candidate Gene for Emotionality in Mice
Source: PLoS One. 2011 Aug 29;6(8):e23604. doi: 10.1371/journal.pone.0023604 (PMC3163650; doi:10.1371/journal.pone.0023604)
Supplement: Table S4 — Primer sequences used for sequencing of the cathepsin B (Ctsb), metallothionein 1 (Mt1) genes as well as for the fragments analyzed by qPCR including the PCR fragment length resulting from each reaction. (DOC) [file pone.0023604.s004.doc]

**Table S4**

| **Gene symbol and primer number** | **Orientation** | | **Primer sequence**  **5’🡪3’** | | **Product size [bp]** | |
| --- | --- | --- | --- | --- | --- | --- |
| *Ctsb* 1 | | forward | | ACA GCA AGG AAC AAC ATA GCA C | | 502 |
|  | | reverse | | GAT GGA AGC AGA AAG GTC AAA G | | |
| *Ctsb* 2 | | forward | | ATA GGT CAT TGG GCT GTG TAG G | | 621 |
|  | | reverse | | GAG AGA CAA GAA CCC AGA AGT ACC | | |
| *Ctsb* 3 | | forward | | GTG AGC AGG CAG TGA TAT GG | | 554 |
|  | | reverse | | AAA TGA GCA GCC TTT CTT GG | | |
| *Ctsb* 4 | | forward | | GCA GCC AGA GAC ACT TTT GG | | 591 |
|  | | reverse | | CCC ATG AAT TTT GTC CAA GG | | |
| *Ctsb* 5 | | forward | | AGA TCA ACT AGG TCA GCC AGC TTC | | 540 |
|  | | reverse | | AAC TGG TGG TTT GTC TGC TCT CT | | |
| *Ctsb* 6 | | forward | | TGC ATG TCA CGA AGA TGT TG | | 562 |
|  | | reverse | | ACT GGA AAG AAG CCG ATC AC | | |
| *Ctsb* 7 | | forward | | TCC ACC TTA ACG CTG ACT CTT C | | 603 |
|  | | reverse | | CTC GCT CCA AAG CTC ACT TAT C | | |
| *Ctsb* 8 | | forward | | ATT GCT CTC CAG TCT CCA TGT T | | 529 |
|  | | reverse | | TCC CTA CAC TCC AAC ACT AGC A | | |
| *Ctsb* 9 | | forward | | AGG CTG GAC GCA ACT TCT AC | | 516 |
|  | | reverse | | CAA TCT TCT CCC ACC TTT CTT G | | |
| *Ctsb* 10 | | forward | | TCA AAT CAG GCA AGG CAT AG | | 532 |
|  | | reverse | | CGG AGG TCA GAG GGA TTA TTA G | | |
| *Ctsb* 11 | | forward | | CTG GAG AGA TGG CTA AGT GGT T | | 606 |
|  | | reverse | | GCA CTG GCT CTA TGC TCA TTT A | | |
| *Ctsb* 12 | | forward | | AGG AAG GAA GGA AGG AAG GAA C | | 581 |
|  | | reverse | | ACA GTG ATG GGA AGA AAT GGA C | | |
| *Ctsb* 13 | | forward | | GCA TAT CTA GGG AGG GAC CAG | | 575 |
|  | | reverse | | AGA GCC TTC AAC CTT CTG AGT G | | |
| *Ctsb* 14 | | forward | | ATC TGC CTT GGA ATT TGC TC | | 537 |
|  | | reverse | | TTG GAG ACG ACA GTT CTT TCT G | | |
| *Ctsb* 15 | | forward | | AGA TGG AGC TTG GTT GAG TCC | | 531 |
|  | | reverse | | AGG GAT GGT GTA TGG TAA GCA G | | |
| *Ctsb* 16 | | forward | | CCT CAA TAC AGG AGC TGA CC | | 545 |
|  | | reverse | | TGA GAC AAG ACA GAG TGT GGA C | | |
| *Ctsb* 17 | | forward | | AAA CAG GAG CAG TAA GGA GGA G | | 573 |
|  | | reverse | | GAA GAG AGC AGA AGG GAG ACT G | | |
| *Ctsb* 18 | | forward | | AAA GAC TAT GGG TGC TGG AGA C | | 515 |
|  | | reverse | | AGT TCT CGT CAC ATG CTG CTC | | |
| *Ctsb* 19 | | forward | | CCC CTC CTC AAA CTA CAT AAG C | | 537 |
|  | | reverse | | CTC CCC TGT CTA CCT CAT TCC | | |
| *Ctsb* 20 | | forward | | CTG CTT TGA CTT CAT TGT CCA G | | 515 |
|  | | reverse | | AAT GTC GAT GGA TGC AGA TG | | |
| *Ctsb* 21 | | forward | | ACT TCA CGA GAG GAC AAA TGC | | 545 |
|  | | reverse | | GTG TGA GCA GTT ACA GGT ACG G | | |
| *Ctsb* 22 | | forward | | GGA ATG TCT GTG CCA ATA AAC C | | 533 |
|  | | reverse | | GCC TCA AAC CGA GTT ACA CTT C | | |
| *Ctsb* 23 | | forward | | TGT AAC TCG GTT TGA GGC AAC | | 526 |
|  | | reverse | | GAG GAC CAC ACA AAG AAC ACA C | | |
| *Ctsb* 24 | | forward | | CCA TGT CGG CAA TCA GAA C | | 581 |
|  | | reverse | | ACC ACA CAA AGA ACA CAC AAC G | | |
| *Ctsb* 25 | | forward | | CCG ATC TCT GGG AGT TTG AG | | 488 |
|  | | reverse | | GAT GTG CTT GCT ACC TTC CTC T | | |
| *Ctsb* 26 | | forward | | CGC TCT CAC TTC CAC TAC CAC | | 591 |
|  | | reverse | | CTA AGT TCA ATT CCC AGC AAC C | | |
| *Mt1* 1 | | forward | | CAA GAG GTC TAA AGG CCC AAG | | 511 |
|  | | reverse | | TGG TCA ACA AAG TGA GTT CCA G | | |
| *Mt1* 2 | | forward | | GAT CTG GAG AGA ACT GAC CAA C | | 564 |
|  | | reverse | | TTC ATG GTG GTT TAG ATA CAA GTG | | |
| *Mt1* 3 | | forward | | GCT GTG TTG TCT CCT CCA AG | | 601 |
|  | | reverse | | TGC ATA CCA TCA CTT CTG AGC | | |
| *Mt1* 4 | | forward | | CTG AAT CCT CTG TCC TTG TGT G | | 535 |
|  | | reverse | | CTC TCT CTG GAT CGA AGC TAG G | | |
| *Mt1* 5 | | forward | | AGC AGA TGG GTT AAG GTG AGT G | | 546 |
|  | | reverse | | CTG CCC TCT TTA TAG TCG TTG G | | |
| *Mt1* 6 | | forward | | TGA CTA TGC GTG GGC TGG AG | | 659 |
|  | | reverse | | CAT GAG GGA GGC AGC ATT ACA G | | |
| *Mt1* 7 | | forward | | CCC TGA CTT AAC CTG TGA GGA G | | 585 |
|  | | reverse | | GGG TGG AAC TGT ATA GGA AGA CG | | |
| *Mt1* 8 | | forward | | TCC TTT CTA GGC TGC TGG CTC | | 562 |
|  | | reverse | | TAC CCA CCT CCT TAT ACC CAA C | | |
| *Mt1* 9 | | forward | | TGG TCA GGT CTT GTG TTA GGG | | 642 |
|  | | reverse | | GGC CAT CTT CTG CTA CAT ACG | | |
| *Mt1* 10 | | forward | | TCA GCT ATT GGA TGG AAC ACA G | | 629 |
|  | | reverse | | GAG ATG GCT CAG TGG GTA AGA G | | |
| *Mt1* 11 | | forward | | ATT TGA ACT CCT GAC CTT CG | | 640 |
|  | | reverse | | ACG TCT CAC GGG CTA GAG | | |
| *Stx3* 1 | | forward | | ATC AGG GAG CCA TGA TTG AC | | 492 |
|  | | reverse | | CAG GCT GTG TGT GTG TTG TG | | |
| *Stx3* 2 | | forward | | ATT CCC TGT CCC TTC TGT CC | | 297 |
|  | | reverse | | CCT CAC CCC CTC CTT ATT GT | | |
| *Abca2* | | forward | | TTT GCG AAG AAG CAG AGT GA | | 531 |
|  | | reverse | | CGC TGC AGA GAA GAG TCA CA | | |
| *Enpp5* | | forward | | GAC CGC TAC CTG GAC AAA GA | | 506 |
|  | | reverse | | TTG TCA CTC CCA AGG AGG AG | | |
| *Zfp672* | | forward | | GTT ATC CCA GGC TGC AGG TA | | 415 |
|  | | reverse | | GGA AGT TGC CCA AGG AAA GT | | |
| *Coro7* | | forward | | CGG CTT CTC TTC ATT CAC CT | | 409 |
|  | | reverse | | CTT CTC CAG TGC CAG ACA GA | | |
| *Pdhb* | | forward | | AGT TGC CCA TTC AAG ACC AG | | 593 |
|  | | reverse | | TCC AGT GAA AGA GGG CAA AT | | |
| *Hmgn3* | | forward | | AAC TAA GCA GGA GCC CAC AA | | 490 |
|  | | reverse | | CGG AAT GCT GAA TCC ATT TC | | |
| *Slc25a17* | | forward | | GCT TGA GAC GGT CCT TTC AC | | 300 |
|  | | reverse | | ATT GGA GCA GCA GGA CTG G | | |
| *Mmp15* 1 | | forward | | ACC CAG TGC CCT CTT AAC CT | | 425 |
|  | | reverse | | ACT CGG ACT CAC GTT TTC GT | | |
| *Mmp15* 2 | | forward | | CGC TGC CTG GAA GAG ATA AC | | 347 |
|  | | reverse | | CTG GGG TCA GAT AAG GAC CA | | |
